# Structural elucidation of succinimide-derived hydrolytic isomers of atosiban using a conventional CID-based Asp/IsoAsp discrimination strategy

**DOI:** 10.1039/d6ra03337g

**Published:** 2026-07-06

**Authors:** Xin Lu, Qianqian Wang, Haijiao Bai, Zengli Li, Hu Liu, Congming Bian, Xiaojie Han, Pei Wang

**Affiliations:** a Tianjin Institute for Drug Control Tianjin 300070 China ritaw_81@163.com; b State Key Laboratory of Medicinal Chemical Biology, Nankai University Tianjin 300350 China; c Anhui Anke Biotechnology (Group) Co., Ltd Hefei 230088 China

## Abstract

The succinimide-mediated degradation system of atosiban generates four hydrolytic impurities that share identical mass shifts and modification sites, posing a significant analytical challenge in peptide drug development. In this study, a discrimination strategy for Asp/isoAsp isomers was developed under conventional collision-induced dissociation (CID) conditions by exploiting subtle differences in MS/MS fragmentation patterns, in combination with the oxazolone-mediated b-ion formation mechanism. The observed fragmentation behavior is mechanistically rationalized by the conformational constraint of isoAsp residues in the gas phase. This study demonstrates, for the first time, that Asp and isoAsp impurities in singly charged peptide ions can be differentiated using only widely accessible CID techniques, without reliance on high charge-state fragmentation methods such as electron transfer dissociation (ETD) or electron capture dissociation (ECD), or specialized instrumentation such as electron activated dissociation (EAD). This significantly enhances the practical feasibility of the method in industrial quality control settings. The D/L configurations of the hydrolytic products were assigned based on an α-carbon carbanion-mediated racemization mechanism, supported by hydrolysis experiments under acidic and basic conditions. As a result, complete structural elucidation of four hydrolytic impurities with identical mass and modification sites was achieved. This work provides a practical and mechanistically grounded analytical strategy for the structural characterization of succinimide-derived impurities in peptide drugs, with potential applicability to other peptide systems sharing similar structural features.

## Abstract 2

The succinimide-mediated degradation system of atosiban generates four hydrolytic impurities that share identical mass shifts and modification sites, posing a significant analytical challenge in peptide drug development.

## Graphical abstract

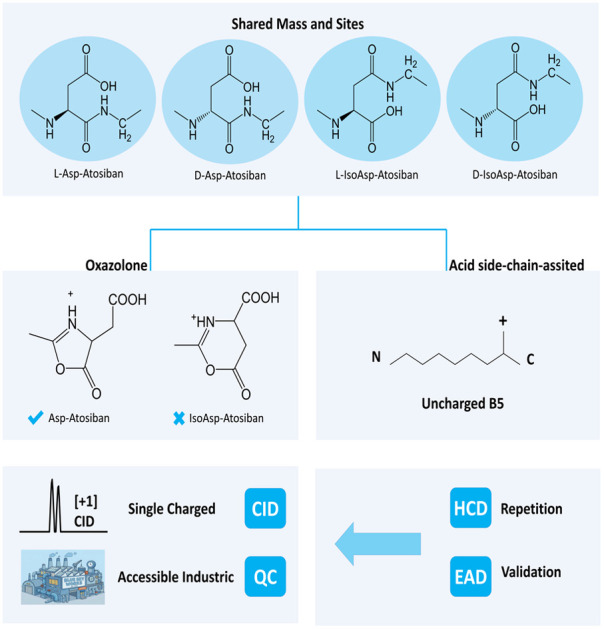

## 1. Introduction

According to regulatory guidelines from the U.S. Food and Drug Administration (FDA) and the European Medicines Agency (EMA), structural identification is required when an individual unknown impurity in peptide drugs exceeds the identification threshold (typically 0.5%).^1–3^ Elucidation of impurity structures is not only a fundamental requirement for regulatory submission, but also a prerequisite for the synthesis of reference standards and subsequent pharmacological and toxicological evaluations. Therefore, the development of efficient and reliable strategies for peptide impurity characterization is of critical importance in both innovative and generic drug development.

In the vasopressin/oxytocin peptide family, a conserved pharmacophoric structural motif is formed by the combination of an asparagine (Asn) residue located in the middle of the sequence and a basic amino acid near the C-terminus. In atosiban and related vasopressin/oxytocin peptides, the Asn residue (Asn5 in atosiban) is positioned at a key turn within the cyclic structure and plays an essential role in modulating the spatial orientation between the cyclic core and the C-terminal tail through intramolecular hydrogen bonding. Meanwhile, the C-terminal basic residue serves as a “charge anchor” for receptor binding, thereby determining agonistic or antagonistic activity. This structural combination is highly conserved among peptides such as terlipressin, vasopressin, desmopressin, atosiban, and barusiban, and can be regarded as a structural fingerprint of this peptide class.^4–7^

The Asn residue within this motif is prone to succinimide-mediated degradation, a well-recognized pathway in peptide pharmaceuticals and protein therapeutics.^8,9^ Specifically, Asn undergoes deamidation *via* intramolecular cyclization to form a five-membered succinimide intermediate, which can subsequently hydrolyze to yield two types of products: aspartic acid (Asp) and isoaspartic acid (isoAsp). Hydrolysis of the succinimide ring may occur at different positions (see Fig. S1), and is often accompanied by racemization at the chiral center, ultimately generating four distinct hydrolytic products: D-Asp, L-Asp, D-isoAsp, and L-isoAsp.^10–13^ These four hydrolytic impurities exhibit highly similar polarity and chromatographic behavior, making them extremely difficult to separate. Moreover, since they share identical mass shifts and modification sites, no differences are expected at the level of theoretical fragment masses, rendering them indistinguishable under conventional CID-based mass spectrometry. This degradation system is widely observed in vasopressin/oxytocin peptides and represents a well-recognized analytical challenge in peptide quality studies.

In industrial quality control settings for peptides, CID is the most commonly employed fragmentation technique as its fragmentation mechanism has been extensively investigated in peptide mass spectrometry research.^14,15^ However, as noted above, the identical mass changes and modification sites of these hydrolytic products make them intrinsically difficult to distinguish under conventional CID conditions. Advanced fragmentation techniques such as electron transfer dissociation (ETD) or electron capture dissociation (ECD) have been reported to provide diagnostic ions for differentiating Asp and isoAsp in certain systems. However, these approaches typically require multiply charged precursor ions and specific instrumentation, limiting their applicability to singly charged species.^16,17^ Electron activated dissociation (EAD) has demonstrated the capability to distinguish Asp and isoAsp even in singly charged peptides,^18,19^ but its implementation relies on specialized and costly high-resolution mass spectrometers, resulting in limited accessibility in routine industrial laboratories. In practice, structural elucidation often relies on proposing multiple candidate structures, synthesizing reference standards, and confirming identities by comparing chromatographic retention times. However, this approach is time-consuming, has a low success rate, and provides limited structural specificity. Therefore, the development of a CID-based strategy capable of differentiating Asp and isoAsp under conventional conditions would offer significant practical value for industrial applications.

Atosiban is a synthetic nonapeptide oxytocin receptor antagonist that is clinically used to suppress preterm uterine contractions. Compared with β-adrenergic agonists, it exhibits a more favorable safety profile and maintains stable clinical demand.^20–22^ With the expiration of the original patent, atosiban injection has become a highly active target in generic peptide drug development, with a growing number of manufacturers entering this field. Structurally, atosiban contains an Asn5 and an Orn8, with an amidated C-terminus. As a parenteral formulation, the drug product is particularly susceptible to hydrolytic degradation. The impurity profile of atosiban has been investigated in previous studies.^23,24^ Notably, the Asn residue readily undergoes succinimide-mediated degradation, generating a series of deamidation and hydrolysis products, some of which exceed the identification threshold. Within the molecule, only the ornithine residue provides a basic side chain, which strongly favors protonation at this site under positive electrospray ionization (ESI) conditions. As a result, atosiban predominantly forms singly charged precursor ions, making it difficult to apply fragmentation techniques such as ETD or ECD that rely on higher charge states for effective Asp/isoAsp differentiation. Therefore, the establishment of a reliable structural elucidation strategy for the succinimide-mediated degradation system of atosiban is of both direct industrial relevance and broader analytical significance. In particular, such a strategy may be extendable to other vasopressin/oxytocin peptides that share the structural motif of a centrally located Asn residue and a C-terminal basic amino acid.

In this study, the degradation pathway was first characterized using high-resolution mass spectrometry. Subsequently, the MS/MS fragmentation behavior of the hydrolytic impurities was systematically investigated under conventional CID conditions. Subtle yet reproducible differences in fragment ions were observed. Based on the oxazolone-mediated b-ion formation mechanism and the acid side-chain-assisted cleavage pathway, the conformational differences between Asp and isoAsp residues in singly charged gas-phase ions were analyzed. The experimental observations suggest that the formation of diagnostic ions is substantially suppressed in isoAsp-containing species, likely due to conformational constraints that reduce the accessibility of productive oxazolone-mediated cyclization under CID conditions.

We demonstrate that under specific structural constraints, particularly charge localization within singly protonated peptide ions (*e.g.*, in the vasopressin/oxytocin peptide family), widely accessible CID fragmentation can exhibit differential accessibility toward oxazolone-mediated cyclization pathways. Rather than introducing a new fragmentation mechanism itself, this study reveals a previously underappreciated condition under which CID fragmentation behavior becomes sensitive to Asp/isoAsp structural differences. This discrimination framework is free from the constraints imposed by high charge states and instrument-specific requirements.

Furthermore, by synthesizing the L-succinimide intermediate and conducting hydrolysis experiments under different pH conditions, the complete assignment of both Asp/isoAsp isomers and their corresponding D/L configurations was achieved. The use of conventional CID fragmentation provides a practically accessible solution for industrial quality control. Given the ongoing expansion of generic development within the vasopressin/oxytocin peptide family, the strategy proposed in this work offers substantial industrial relevance. Moreover, the shared structural characteristics of this peptide class provide a foundation for the potential extension of this mechanism-driven Asp/isoAsp discrimination strategy to related systems. It should be noted that the present CID-based strategy is intended primarily for qualitative structural discrimination and mechanistically supported assignment of Asp/isoAsp isomers, rather than quantitative determination based on diagnostic fragment ion intensities.

## 2. Experimental

### 2.1. Chemicals and materials

Atosiban active pharmaceutical ingredient (API) and injection samples were provided by Anhui Anke Biotechnology Co., Ltd (Hefei, China). Trifluoroacetic acid (TFA), formic acid (FA), and acetonitrile (ACN) were all of mass spectrometry grade and purchased from Merck. Experimental water was prepared using a Milli-Q purification system. Hydrochloric acid and sodium hydroxide were of analytical grade and were used to adjust the pH conditions in hydrolysis experiments. The L-succinimide intermediate used for mechanistic studies was synthesized *via* solid-phase synthesis by Anhui Anke Biotechnology Co., Ltd.

### 2.2. HPLC method

Liquid chromatographic analysis was performed using a high-performance liquid chromatography system (Thermo Fisher Scientific Ultimate 3000) equipped with an Inertsil ODS-3 column (250 × 4.6 mm, 5 µm). The mobile phase A consisted of an aqueous solution of trifluoroacetic acid (pH 3.2), and mobile phase B was a mixture of 60% acetonitrile and 40% methanol. Separation was achieved using a gradient elution program as follows: 27% B (0–12 min), 27–32% B (12–25 min), 32–36% B (25–36 min), 36–54% B (36–41 min), 54% B (41–46 min), 54–27% B (46–46.1 min), and 27% B (46.1–70 min). The flow rate was set at 1.0 mL min^−1^, the column temperature was maintained at 45 °C, the injection volume was 25 µL, and the detection wavelength was set at 220 nm.

### 2.3. UPLC–MS/MS (CID)

Method (CID) chromatographic separation was performed using an ultra-performance liquid chromatography system (Waters ACQUITY) equipped with a Waters ACQUITY UPLC BEH C18 column (2.1 mm × 100 mm, 1.7 µm). The mobile phase A consisted of 0.1% formic acid in water, and mobile phase B consisted of 0.1% formic acid in acetonitrile. Separation was achieved using a gradient elution program as follows: 5% B (0–2 min), 5–35% B (2–18 min), 35–80% B (18–22 min), 80% B (22–25 min), and 80–5% B (25–30 min). The flow rate was 0.3 mL min^−1^, the column temperature was maintained at 40 °C, and the injection volume was 5 µL.

Mass spectrometric detection was performed using a Waters Xevo G2 XS QTOF mass spectrometer equipped with an electrospray ionization (ESI) source operating in positive ion mode. The capillary voltage was set at 3.0 kV, and the cone voltage was set at 35 V. The source temperature was maintained at 120 °C, and the desolvation temperature was set at 350 °C, with a desolvation gas flow of approximately 600 L h^−1^. The full scan mass range was set to *m*/*z* 100–1500. MS/MS experiments were conducted using collision-induced dissociation (CID) with a collision energy of 14–47 eV.

### 2.4. UPLC-MS/MS method (HCD)

Details were shown in the SI.

### 2.5. UPLC-MS/MS method (EAD)

Details were shown in the SI.

### 2.6. Hydrolysis experiments

The synthesized succinimide intermediate was subjected to hydrolysis under both acidic and basic conditions. For the acidic hydrolysis experiment, the L-succinimide intermediate was dissolved in a 10 mM ammonium acetate solution (pH 6.0) and incubated at room temperature for 24 h. For the basic hydrolysis experiment, the sample was dissolved in a 10 mM ammonium acetate solution (pH 8.0) and treated under the same temperature and time conditions.

After the reactions, the samples were directly analyzed by LC-MS to compare the compositional differences of the hydrolysis products under different conditions.

### 2.7. Density functional theory (DFT) calculations

Quantum chemical calculations were performed using Gaussian 16 software. All calculations were carried out in the gas phase at the B3LYP/6-31+G(d,p) level of theory. The QST2 method was employed to local transition state, and the connectivity of the reaction pathways was verified by intrinsic reaction coordinate (IRC) calculations. The activation free energies (Δ*G*^‡^) and ZPE-corrected relative energy profiles of the key cyclization pathways were obtained from these calculations.

## 3. Results and discussion

### 3.1. Identification of degradation impurities exceeding the qualification threshold in stability samples

**Fig. 1:**
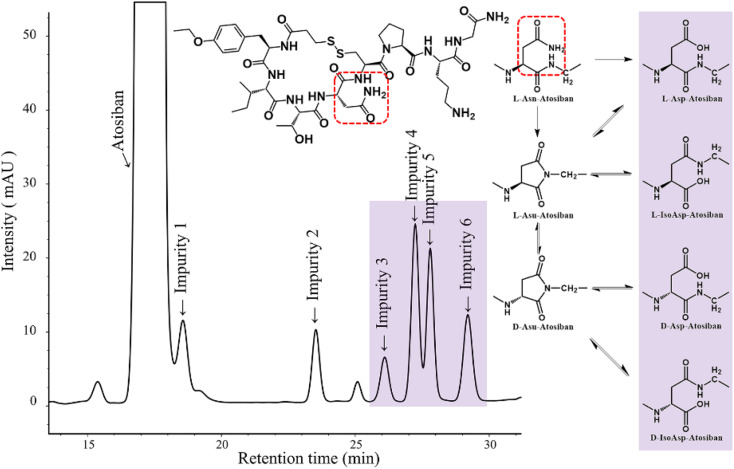
Related-substances chromatogram of atosiban injection within shelf life. The major peak at 15.5 min corresponds to atosiban. The chemical structure of atosiban and the Asn5-associated succinimide-mediated degradation pathway are also illustrated.

During stability studies of atosiban injection, six impurities were consistently detected across multiple batches, with their levels increasing over time. The impurity contents in samples within the shelf life reached 0.41%, 0.27%, 0.18%, 0.66%, 0.53%, and 0.38%, respectively, with several exceeding the identification threshold specified in the European Pharmacopoeia. The corresponding chromatogram is shown in Fig. 1. According to EMA regulatory requirements, structural identification of these impurities is necessary.

### 3.2. Confirmation of the succinimide-mediated degradation pathway by high-resolution mass spectrometry

**Fig. 2:**
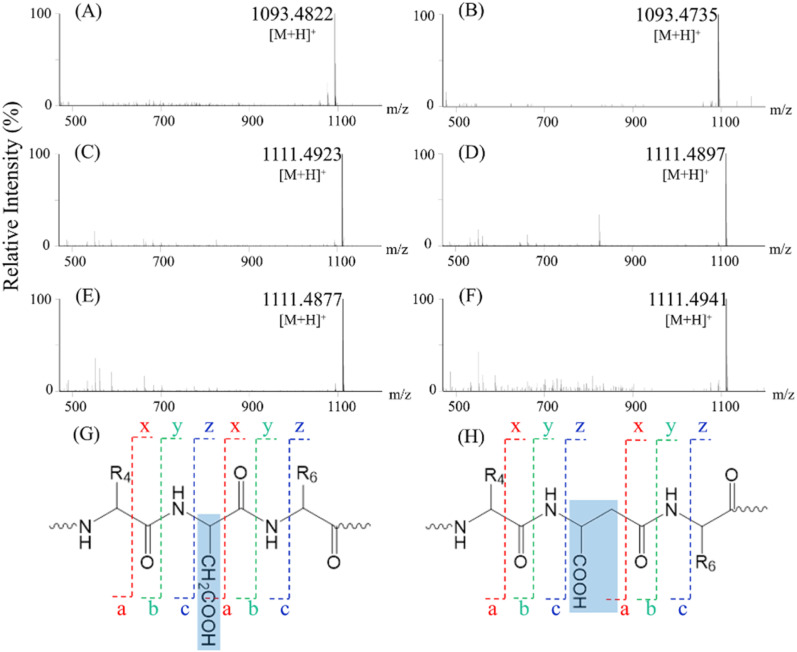
High-resolution MS spectra of the six impurities acquired in positive ESI mode ([M + H]^+^). Panels A–F correspond to Impurities 1–6, with mass deviations from the theoretical values of 4.3 ppm, 3.6 ppm, 1.1 ppm, 1.3 ppm, 3.1 ppm, and 2.7 ppm, respectively. Schematic comparison of theoretical CID fragmentation patterns of Asp-containing (G) and isoAsp-containing (H) peptide structures. The conserved C_3_H_4_O_2_ moiety is retained within corresponding fragment ions, resulting in theoretically identical fragment masses.

High-resolution mass spectrometry (HRMS) was employed to determine the monoisotopic masses of the six impurities. Two impurities exhibited a mass decrease of 17.02 Da relative to the main component, consistent with the theoretical mass loss (−17.0265 Da) associated with Asn deamidation *via* succinimide formation. The remaining four impurities showed a mass increase of 0.98 Da, corresponding to the theoretical mass gain (+0.9840 Da) resulting from subsequent hydrolysis of the succinimide intermediate to Asp or isoAsp residues. The MS spectra are presented in Fig. 2. These mass changes are characteristic of the well-established Asn succinimide degradation pathway.

The atosiban molecule contains two amide functionalities: the side-chain amide of the Asn residue at position 5 and the amidated C-terminus. Both are, in principle, susceptible to hydrolysis. To determine the modification site, CID MS/MS analysis was performed on all six impurities. The results showed that all hydrolytic changes were localized to the residue at position 5, while the C-terminal structure remained intact. These findings are consistent with the assignment of the six impurities as a complete Asn succinimide-mediated degradation system, including two deamidated succinimide intermediates and four hydrolytic products (Asp/isoAsp and their respective D/L configurations).

### 3.3. Emergence of differential CID fragmentation behavior under charge-localized conditions

In principle, peptide backbone fragmentation under conventional CID conditions predominantly yields b- and y-type ions, with occasional formation of a/*x* or c/z ions. In all these fragmentation pathways, the C_3_H_4_O_2_ moiety derived from Asp or isoAsp is retained within the same fragment ion, making it theoretically impossible to distinguish between the two isomers based solely on fragment masses. Theoretical fragmentation patterns are illustrated in Fig. 2.

It should be noted that advanced fragmentation techniques such as electron transfer dissociation (ETD) or electron capture dissociation (ECD) can, in certain systems, provide diagnostic ions (*e.g.*, *c* +57/*z* −57) for differentiating isoAsp residues. However, these methods typically require multiply charged precursor ions and specialized instrumentation, limiting their applicability in routine industrial quality control settings. Therefore, this study focuses on exploring whether diagnostic differences between isomers can be identified under conventional CID conditions.

CID MS/MS spectra were acquired for the four hydrolytic impurities. Overall, the spectra appeared highly similar, with no obvious differences observed at the global level (Fig. S2A), consistent with theoretical expectations. However, upon careful inspection of low-abundance fragments in expanded mass regions, subtle but reproducible differences were identified. Specifically, in the *m*/*z* range of 660–670, a fragment ion at *m*/*z* 666 was consistently observed for impurities 3 and 5, whereas this ion was absent for impurities 4 and 6 across repeated measurements (Fig. S2B).

In addition, analysis was performed on another high-resolution mass spectrometer using higher-energy collisional dissociation (HCD), which also relies on kinetic-to-internal energy conversion *via* inert gas collisions. Consistent results were obtained, with the presence or absence of the *m*/*z* 666 ion matching the CID observations (see Fig. S3). These findings substantially reduce the likelihood that the observed differences arise from random experimental variation and instead support the interpretation that they originate from intrinsic differences in CID fragmentation accessibility.

### 3.4. Mechanistic rationalization of differential CID fragmentation behavior

Accurate mass analysis indicates that the ion at *m*/*z* 666 corresponds to the theoretical B5 ion, with a calculated *m*/*z* value of 666.2804. This observation suggests that, although Asp and isoAsp are indistinguishable at the level of theoretical fragment masses, their CID fragmentation behaviors may differ in pathway accessibility or fragmentation efficiency.

**Fig. 3:**
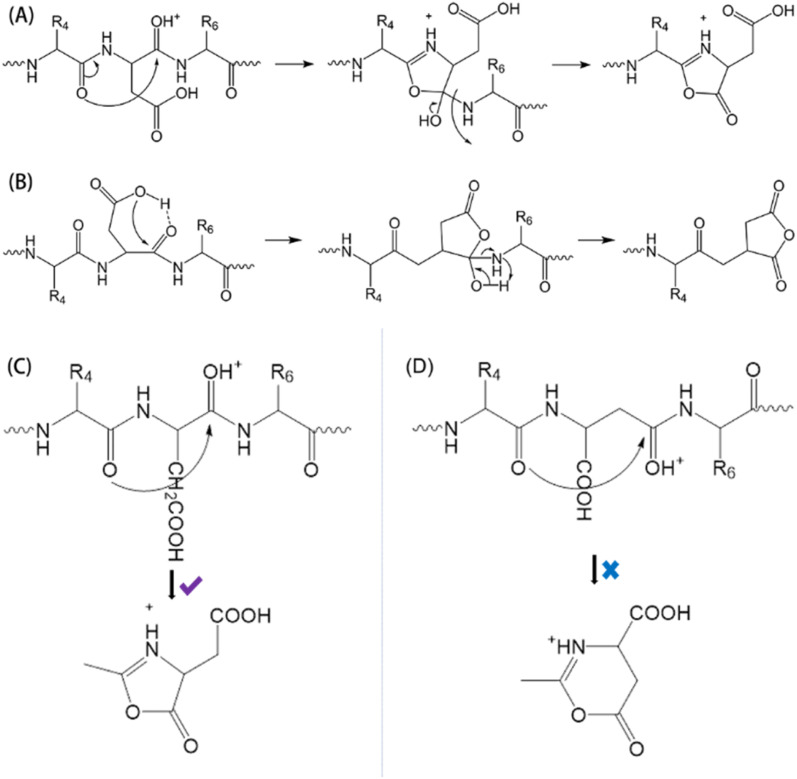
Mechanism of oxazolone-mediated cleavage (A) and acid side-chain-assisted cleavage (B). Comparison of oxazolone-mediated fragmentation mechanisms for Asp and isoAsp residues (C and D).

To elucidate the origin of the *m*/*z* 666 (B5) ion observed in certain impurities but absent in others, it is necessary to first examine its formation mechanism. Under CID conditions, the formation of peptide b-ions is generally described by the classical oxazolone-mediated fragmentation pathway.^25–28^ In this mechanism, the carbonyl oxygen of the amide bond acts as an intramolecular nucleophile, attacking the protonated carbonyl carbon to form a five-membered oxazolone ring intermediate, followed by cleavage of the amide bond with retention of the positive charge on the N-terminal fragment. This five-membered ring exhibits conjugation, which contributes to charge stabilization (Fig. 3A). This pathway is widely accepted as the predominant route for CID b-ion formation in many peptide systems.

In gas-phase CID fragmentation, the formation of b/y ions is widely accepted to proceed predominantly *via* the oxazolone mechanism, while acid side-chain-assisted cleavage represents the only well-established alternative pathway associated with Asp residues.^29,30^ In this alternative mechanism, the side-chain carboxyl group acts as a nucleophile, facilitated by hydrogen bonding, to attack the backbone carbonyl carbon, forming a cyclic intermediate that induces peptide bond cleavage (Fig. 3B). Notably, this pathway leads only to bond cleavage and does not generate a charge-bearing fragment.

As a member of the vasopressin/oxytocin peptide family, atosiban possesses the characteristic structural motif of a centrally located Asn residue and a C-terminal basic amino acid. Specifically, the ornithine residue at position 8 contains a primary amine side chain with high proton affinity under positive ESI conditions, effectively localizing the positive charge. Under such conditions, acid side-chain-assisted cleavage can only produce y-type ions, but not charge-retaining b-ions. Within the current mechanistic framework of peptide CID fragmentation, oxazolone-mediated cleavage and acid side-chain-assisted cleavage are generally regarded as the major pathways relevant to the present system. Therefore, in the present system, the generation of charge-bearing N-terminal b-ions is primarily governed by the oxazolone mechanism.

The feasibility of oxazolone-mediated fragmentation for isoAsp residues was further investigated. For Asp, oxazolone formation proceeds *via* a five-membered ring intermediate. This ring consists of four sp^2^-hybridized atoms and one sp^3^-hybridized atom, forming a nearly planar structure that facilitates intramolecular nucleophilic attack and stabilizes the positive charge through π-conjugation (Fig. 3C).

In contrast, formation of an analogous intermediate for isoAsp would require a six-membered ring transition state. This structure contains two sp^3^-hybridized atoms embedded within a conjugated framework (Fig. 3D), which significantly distorts the planarity of the system and disrupts π-conjugation. This conformational feature increases the activation barrier for cyclization. Combined with the experimental observation that the corresponding b-ion is not detected across a range of collision energies, it can be inferred that this cyclization pathway is kinetically inaccessible under the CID conditions employed in this study. These structural features are expected to substantially reduce the accessibility of productive charge-retaining oxazolone cyclization in isoAsp-containing species under the CID conditions employed, consistent with the experimentally observed suppression of B5 ion formation.

Based on the mechanistic analysis and experimental observations, impurities 3 and 5 are therefore interpreted as Asp-type hydrolysis products, whereas impurities 4 and 6, in which the B5 ion is absent, are assigned as isoAsp-type products.

To further evaluate the formation behavior of the diagnostic B5 ion, energy-resolved CID MS/MS experiments were performed over a wide collision-energy range. For Asp-type impurities (Impurities 3 and 5), the B5 ion (*m*/*z* 666) first became detectable at approximately 17.2 eV, increased progressively with increasing collision energy, reached maximal abundance at intermediate energies, and subsequently decreased at higher energies until disappearing (Fig. S4 and Table S1). In contrast, no detectable B5 ion was observed for the isoAsp-type impurities (Impurities 4 and 6) throughout the entire collision-energy range investigated. The bell-shaped energy dependence observed for Impurities 3 and 5 is consistent with a sequential fragmentation process in which the B5 ion acts as an intermediate fragment. At low collision energies, insufficient internal energy is available for efficient formation of the B5 ion, whereas at elevated collision energies the B5 ion undergoes further secondary fragmentation. Importantly, the complete absence of the B5 ion in Impurities 4 and 6 across all collision energies provides additional experimental support for the proposed suppression of charge-retaining oxazolone formation in isoAsp-containing species.

Further analysis indicates that the validity of this discrimination strategy depends on two key structural conditions: (i) the presence of an Asn residue susceptible to succinimide formation within the sequence, and (ii) the existence of a basic site capable of preferential protonation in the gas phase, thereby constraining charge distribution during fragmentation. When these conditions are satisfied, isoAsp residues are conformationally restricted and unable to form stable charge-retaining oxazolone intermediates, resulting in characteristic suppression of diagnostic b-ion formation. Therefore, this strategy may be applicable, in a qualitative structural characterization context, to peptide systems with similar structural features, particularly those within the vasopressin/oxytocin family.

It should be emphasized that the discrimination strategy proposed here is not intended to represent a universal CID-based solution for all Asp/isoAsp-containing peptides. The observed differentiation behavior appears to depend strongly on specific structural features of the peptide system, particularly the presence of a localized protonation site and restricted charge mobility within singly protonated precursor ions. Under these conditions, subtle conformational differences between Asp and isoAsp residues may become amplified at the level of CID fragmentation accessibility. Therefore, the present work should be viewed as establishing a structure-dependent analytical framework rather than a universally applicable fragmentation rule.

The present strategy is primarily intended for qualitative structural discrimination rather than quantitative analysis. Since CID fragmentation efficiencies can be influenced by precursor ion conformation, proton mobility, collision energy, and instrument-dependent conditions, the relative intensities of diagnostic fragment ions should not currently be interpreted quantitatively without additional validation.

### 3.5. Validation of the isomer discrimination strategy

#### 3.5.1. Computational support for differential cyclization accessibility

To validate the proposed difference in oxazolone-mediated fragmentation behavior, density functional theory (DFT) calculations were performed using Gaussian software. Geometry optimizations and frequency analyses were conducted in the gas phase at the B3LYP/6-31+G(d,p) level. Transition states were located using the QST2 method, and intrinsic reaction coordinate (IRC) calculations were performed to confirm the connectivity of the reaction pathways.

The results indicate that the intramolecular cyclization leading to a five-membered ring intermediate proceeds with a relatively low activation free energy (Δ*G*^‡^ = 75.8 kJ mol^−1^). Although the collision energy in CID cannot be directly equated to internal energy, prior studies on peptide CID fragmentation suggest that reactions with Δ*G*^‡^ < 100 kJ mol^−1^ are generally accessible under CID conditions.^31,32^

In contrast, calculations of the alternative pathway involving formation of a six-membered ring intermediate revealed a substantially higher activation free energy (Δ*G*^‡^ = 295.4 kJ mol^−1^).

These computational results are consistent with the experimentally observed differential fragmentation behavior and support the proposed mechanistic interpretation. The calculated energy profiles (Fig. S5) further support that formation of the five-membered oxazolone intermediate is energetically accessible under CID conditions, whereas the corresponding six-membered cyclization pathway associated with isoAsp remains kinetically unfavorable. ZPE-corrected relative free energies and transition-state-related structures are provided in the SI (Table S2 and Fig. S6).

#### **3.5.2**. Orthogonal validation

Previous studies have demonstrated that electron-activated dissociation (EAD) enables effective differentiation between Asp and isoAsp residues in peptides, even for singly charged precursor ions.^18,19,33^ EAD induces characteristic fragmentation pathways dominated by N–Cα bond cleavage. In the case of isoAsp residues, the presence of a β-linked backbone structure facilitates a specific rearrangement upon electron activation, accompanied by Cα–Cβ bond cleavage and side-chain migration, ultimately generating a diagnostic ion with a mass shift of *c*_5_ +57.028 Da.

Based on this principle, the four target impurities were analyzed using a high-resolution mass spectrometer equipped with EAD capability. The results show that diagnostic *c*_5_ +57.028 Da ions were clearly observed for Impurities 4 and 6, whereas no such ions were detected for Impurities 3 and 5 under identical conditions (Fig. S7). These findings indicate that Impurities 4 and 6 contain isoAsp residues, while Impurities 3 and 5 correspond to Asp isomers. Notably, this assignment is in complete agreement with the conclusions derived from the CID-based fragmentation analysis, providing orthogonal experimental support for the CID-based structural assignments.

Despite its clear advantage in distinguishing Asp and isoAsp residues, EAD typically requires mass spectrometers equipped with dedicated electron-activation modules and is currently implemented only on a limited number of advanced platforms. As a result, its accessibility in routine industrial quality control laboratories remains limited. In contrast, CID represents the most widely implemented fragmentation mode across LC-MS/MS systems, with well-established methodologies and high transferability. Therefore, from a practical standpoint, the development of an Asp/isoAsp discrimination strategy that does not rely on specialized fragmentation techniques such as EAD is of greater industrial relevance. In this context, the CID-based strategy proposed in this study provides a directly implementable solution under standard analytical conditions. Importantly, the purpose of incorporating EAD in this study was not to establish a new EAD-based analytical workflow, but rather to independently assess whether the CID-derived assignments were chemically reasonable.

### 3.6. Differentiation of D/L configurations based on racemization mechanism

**Fig. 4:**
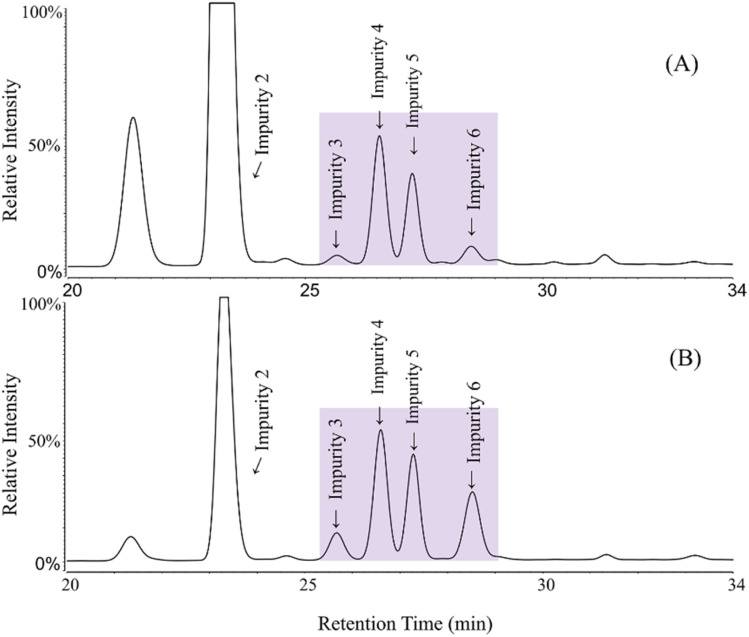
LC chromatograms of hydrolysis products generated from the synthesized L-succinimide intermediate (Impurity 2). Under acidic conditions (A), hydrolysis predominantly produced Impurities 4 and 5. Under basic conditions (B), both racemization and hydrolysis occurred, generating Impurities 3–6. The chromatograms do not contain the atosiban parent compound.

Following the assignment of Asp and isoAsp types, further differentiation of the D- and L-configurations of the four hydrolytic products was required. For this purpose, the synthesized succinimide intermediate (Impurity 2) rather than the atosiban parent compound was used as the reaction substrate in the hydrolysis experiments. Amide hydrolysis can occur under both acidic and basic conditions. To investigate the stereochemical outcome, the L-succinimide impurity was synthesized and subjected to hydrolysis under controlled conditions at pH 2.0 and pH 8.0. Under acidic conditions (pH 2.0), the hydrolysis predominantly generated Impurities 4 and 5 (Fig. 4A). In contrast, under basic conditions, all four impurities (Impurities 3, 4, 5, and 6) were observed (Fig. 4B).

The observed stereochemical outcomes can be rationalized by a carbanion-mediated racemization mechanism. Under basic conditions, the α-hydrogen can be abstracted to form a carbanion intermediate. Three factors favor this process: (i) the bond angle at the chiral center within the five-membered ring is calculated (using VASP software) to be 121.8°, which is close to the 120° characteristic of an sp^2^-hybridized planar structure; (ii) the adjacent carbonyl group exerts an electron-withdrawing effect, stabilizing the carbanion; (iii) deprotonation leads to the formation of an sp^2^-hybridized center that can engage in p–π conjugation with the carbonyl group, promoting planarization and resulting in loss of stereochemical information. Consequently, under basic conditions, the L-succinimide intermediate can undergo racemization *via* a carbanion intermediate, yielding a mixture of D- and L-isomers. In contrast, under acidic conditions, formation of the carbanion is not feasible, and only hydrolysis occurs without racemization.

Based on these observations, Impurities 4 and 5, which are formed under both acidic and basic conditions, are assigned as L-isomers. In contrast, Impurities 3 and 6, which are generated exclusively under basic conditions, are assigned as D-isomers. Through this combined mechanistic and experimental analysis, a mechanistically supported structural assignment of the four hydrolytic impurities—sharing identical molecular weight and modification site—was achieved.

## 4. Conclusions

This study systematically investigated four hydrolytic impurities derived from succinimide-mediated transformation of atosiban, which exhibit identical mass changes and share the same modification site. A CID-based analytical framework was established for differentiating Asp and isoAsp isomers under structurally constrained conditions in singly charged gas-phase ions, based exclusively on conventional collision-induced dissociation (CID) fragmentation. Under structurally constrained and charge-localized conditions, conventional CID fragmentation can exhibit differential accessibility toward Asp- and isoAsp-associated cyclization pathways. The proposed method exploits subtle yet reproducible differences in secondary fragmentation behavior of atosiban-related impurities. Notably, the differentiation is achieved without reliance on high-charge-state fragmentation techniques such as ETD/ECD or specialized electron-based methods such as EAD, but solely through conventional CID. Consistent results obtained under higher-energy collisional dissociation (HCD) further demonstrate the robustness and transferability of the approach across different fragmentation platforms. To support the proposed mechanism, density functional theory (DFT) calculations were performed to evaluate the energy barriers of key intramolecular cyclization pathways. In addition, electron-activated dissociation (EAD) experiments were conducted as an orthogonal validation method, yielding results fully consistent with the CID-based assignments. Considering the limited accessibility of EAD and related techniques in industrial laboratories, the CID-based strategy established in this work provides a more practical and implementable solution for real-world quality control applications. Furthermore, the D/L configurations of the four hydrolytic impurities were successfully differentiated based on a carbanion-mediated racemization mechanism. This integrated analytical approach demonstrates strong applicability in the industrial quality control of atosiban. Given that peptides within the vasopressin/oxytocin family commonly share structural features such as a centrally located Asn residue and a C-terminal basic amino acid, the CID-based discrimination strategy developed herein has potential applicability to a broader class of structurally related peptide therapeutics.

The present findings do not imply universal applicability across all peptide systems, but rather demonstrate that under appropriate structural conditions, conventional CID fragmentation may possess previously underappreciated discriminatory capability toward subtle side-chain isomerization events. This concept provides a mechanism-driven analytical framework for peptide isomer characterization without dependence on high-charge-state fragmentation techniques, offering clear advantages in industrial quality control settings. While the generalizability of this strategy is contingent upon specific sequence features and charge distribution (*e.g.*, the presence of a well-defined protonation site), its applicability remains highly relevant for peptide systems with similar structural characteristics. In particular, given the structural conservation, active development, and industrial significance of vasopressin/oxytocin analogs, the practical value of this approach is both immediate and substantial. Overall, this work expands the analytical capability of conventional mass spectrometry for peptide isomer differentiation and provides a practically accessible and mechanistically rationalized analytical approach for structurally challenging impurity characterization in pharmaceutical development.

## Author contributions

Xiaojie Han and Pei Wang are co-corresponding authors and contributed equally to this article. Xin Lu: data curation, formal analysis, writing – original draft, writing – review and editing, investigation, methodology, visualization, conceptualization. Qianqian Wang: data curation, formal analysis, writing – original draft, writing – review and editing, investigation, methodology, visualization, conceptualization. Haijiao Bai: conceptualization, investigation. Zengli Li: resources, formal analysis. Hu Liu: resources, methodology. Congming Bian: methodology. Xiaojie Han: supervision, resources, funding acquisition, supervision. Pei Wang: supervision, resources, funding acquisition, supervision.

## Conflicts of interest

The authors declare that there are no conflicts of interest.

## Supplementary Material

RA-OLF-D6RA03337G-s001

## Data Availability

All data supporting the findings of this study are available within the paper and its supplementary information (SI). Supplementary information: additional experimental procedures, MS/MS spectra, energy-resolved CID data, DFT calculation results (including ZPE-corrected relative free energies and optimized structures), and supporting figures and tables relevant to this study. See DOI: https://doi.org/10.1039/d6ra03337g.
