# Supplementary material for: Structural elucidation of succinimide-derived hydrolytic isomers of atosiban using a conventional CID-based Asp/IsoAsp discrimination strategy
Source: RSC Adv. 2026 Jul 6;16(36):37713–22. doi: 10.1039/d6ra03337g (PMC13334539; doi:10.1039/d6ra03337g)
Supplement: Supplementary file 1 [file RA-016-D6RA03337G-s001.pdf]

Supplementary Information (SI) for RSC Advances.
This journal is © The Royal Society of Chemistry 2026

Supplementary Information

Structural Elucidation of Succinimide-Derived Hydrolytic Isomers of Atosiban Using a Conventional CID-Based Asp/IsoAsp Discrimination Strategy

Author：Xin Lu [a‡],Qianqian Wang [b‡],Haijiao Bai [a], Zengli Li [c], Hu Liu [c], Congming Bian [c], Xiaojie Han [a*], Pei Wang [a*]

Affiliations: [a] *Tianjin Institute for Drug Control, Tianjin 300070, China;* [b] *State Key Laboratory of Medicinal Chemical Biology, Nankai University, Tianjin 300350, China;*[c] *Anhui Anke Biotechnology (Group) Co., Ltd, Hefei 230088, China*

Corresponding author: ritaw_81@163.com

Materials and Methods

UPLC–MS/MS Method (HCD)

Chromatographic separation was performed using an ultra-performance liquid chromatography system (Thermo Fisher Scientific Vanquish). The chromatographic column and conditions were identical to those described in Section 2.3.

Mass spectrometric detection was carried out using an Orbitrap Exploris 480 mass spectrometer (Thermo Fisher Scientific, USA) equipped with a heated electrospray ionization (HESI) source operating in positive ion mode. The spray voltage was set to 3200 V, with sheath gas at 35 Arb, auxiliary gas at 10 Arb, and sweep gas at 0 Arb. The ion transfer tube temperature was maintained at 320 °C, and the vaporizer temperature was set at 340 °C. In full scan mode, the mass range was set to m/z 200–2000 with a resolution of 60,000. The ddMS^2 experiments were performed at a resolution of 15,000 using higher-energy collisional dissociation (HCD) with a collision energy of 30 eV. The RF lens was set to 50%, and the AGC target was operated in standard mode.

UPLC–MS/MS (EAD)

Chromatographic separation was performed using an Agilent 1290 ultra-performance liquid chromatography system (Agilent Technologies, Santa Clara, CA, USA). The chromatographic column and conditions were identical to those described in Section 2.3.

Electron-activated dissociation (EAD) experiments were conducted on a SCIEX ZenoTOF 7600 mass spectrometer. Samples were introduced into the mass spectrometer via an electrospray ionization (ESI) source operating in positive ion mode. The ion spray voltage was set at approximately 5.5 kV, and the source temperature was maintained at 500 °C. The curtain gas (CUR) was set to 30 psi, while the nebulizer gas (GS1) and auxiliary gas (GS2) were both set to 50 psi. The declustering potential (DP) was set at 80 V. The mass acquisition range was set to m/z 100–1500. The electric beam current was set to 5000 μA, and the kinetic energy was 8 kV. The EAD reaction time was 10 ms, and the Zeno trap function was enabled (Zeno trap on).

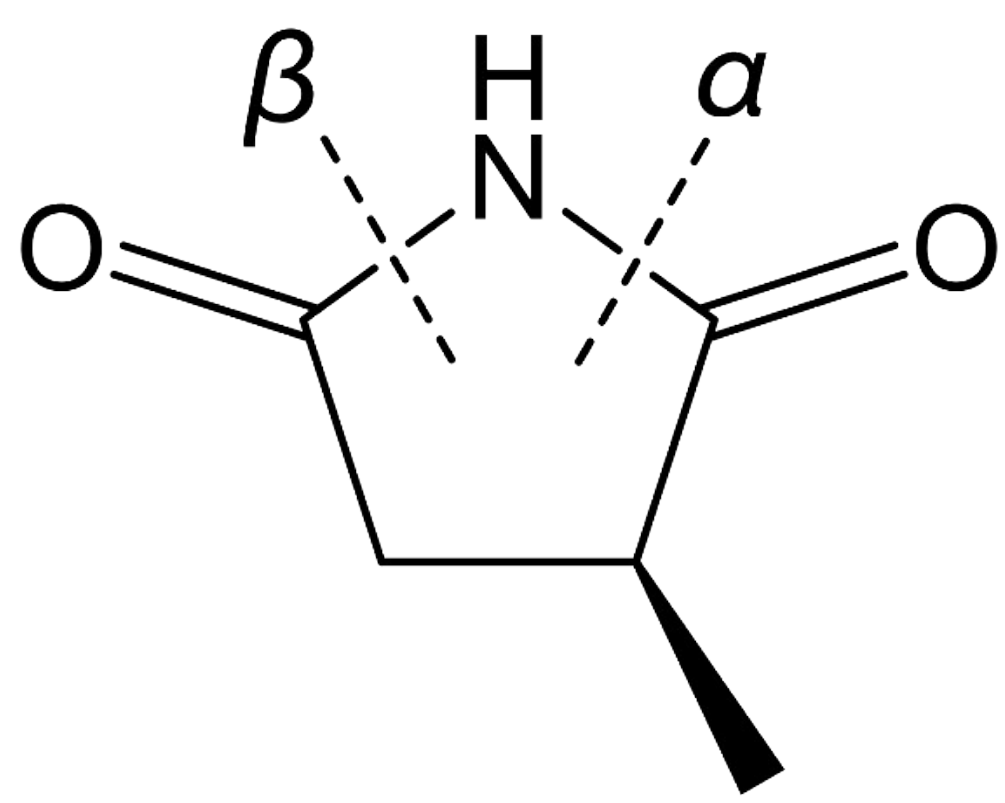

Fig. S1 Different hydrolysis positions of the succinimide ring.

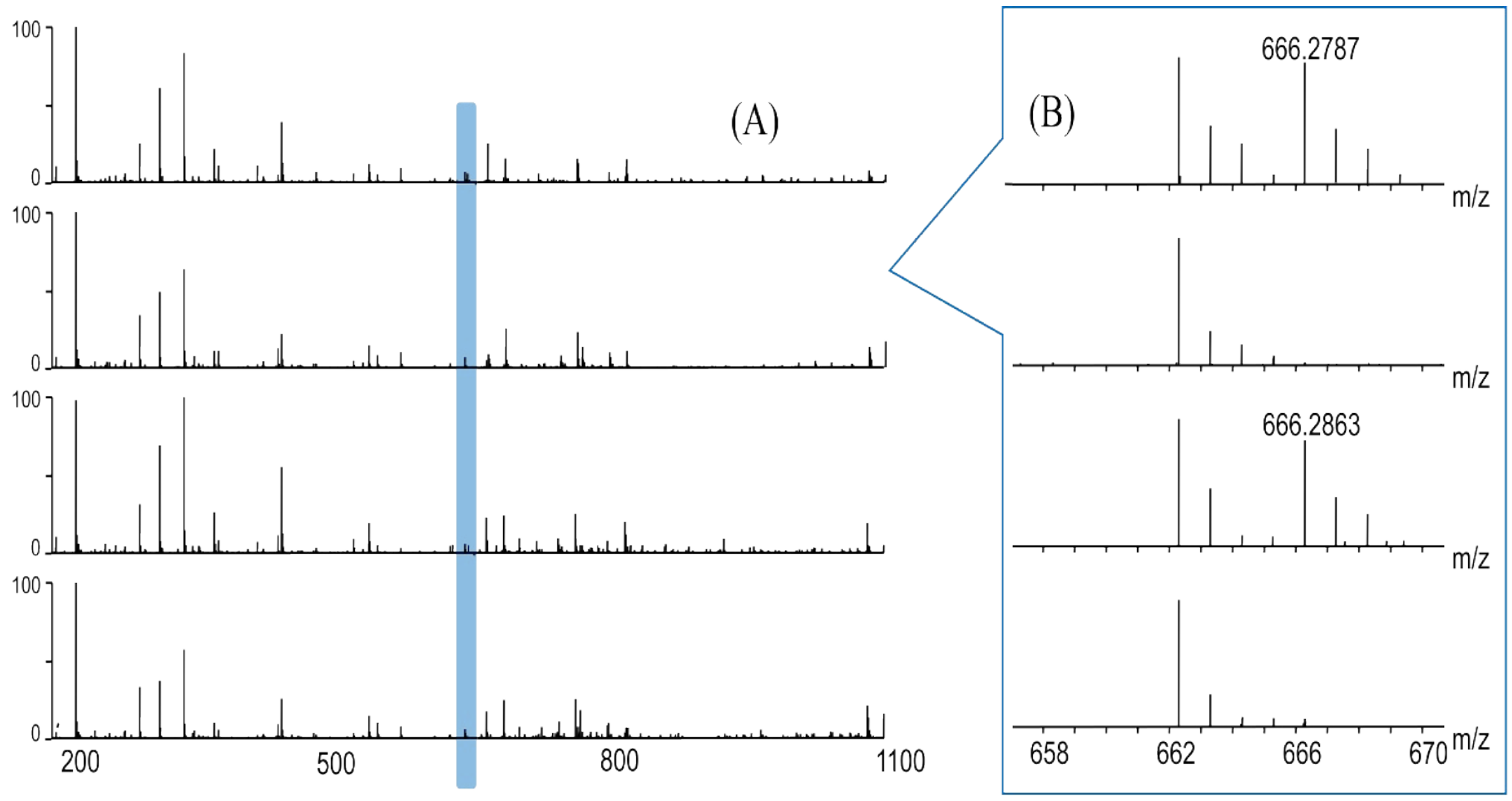

Fig .S2. CID MS/MS spectra of the four hydrolytic impurities and enlarged mass regions highlighting the differential formation behavior of the B5 ion.

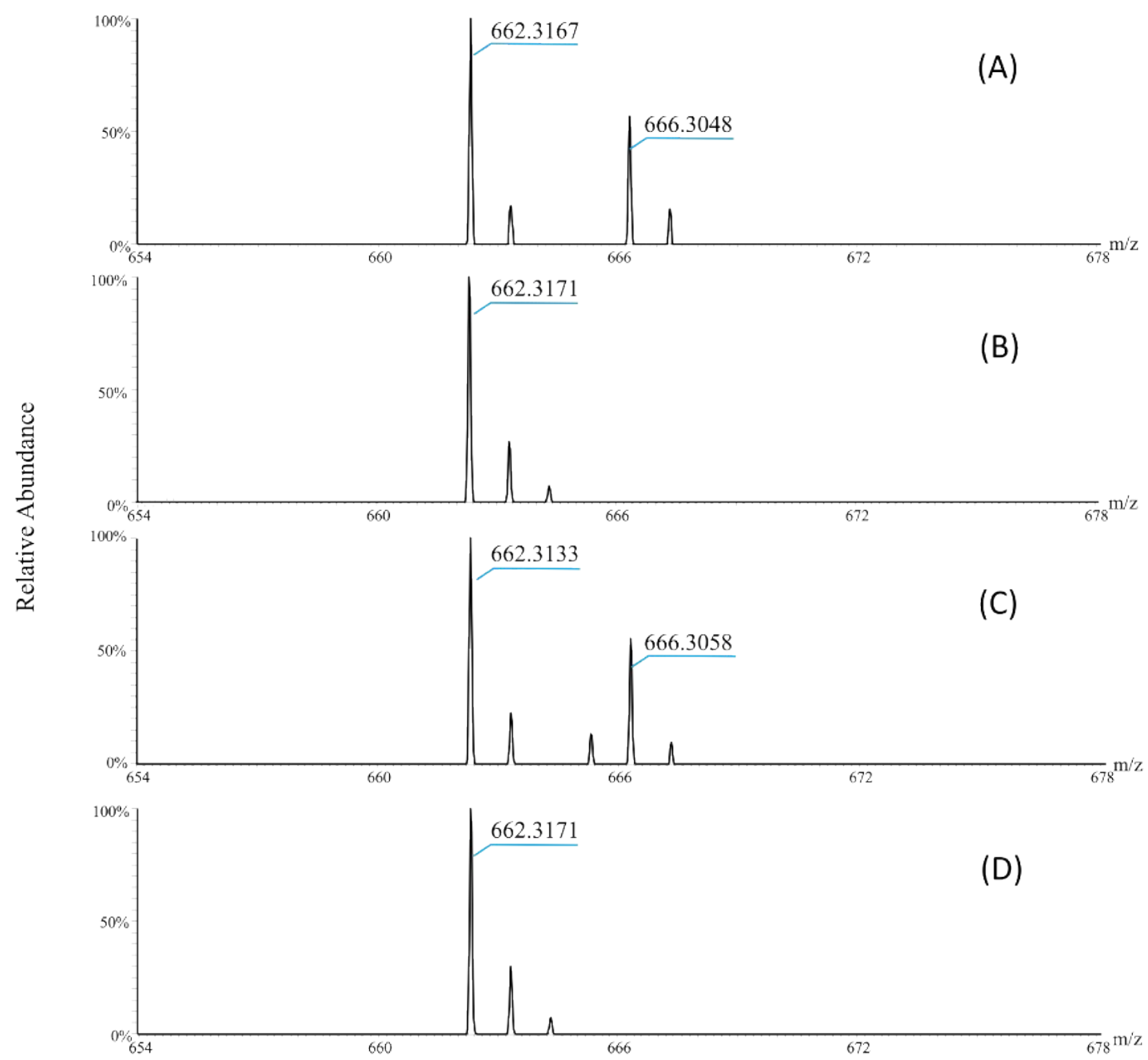

Fig. S3. HCD MS/MS spectra of the four hydrolytic impurities showing the presence or absence of the diagnostic ion at m/z 666, consistent with CID observations. The ion at m/z 666 is observed in Impurities 3 (A) and 5 (C), but is absent in Impurities 4 (B) and 6 (D)

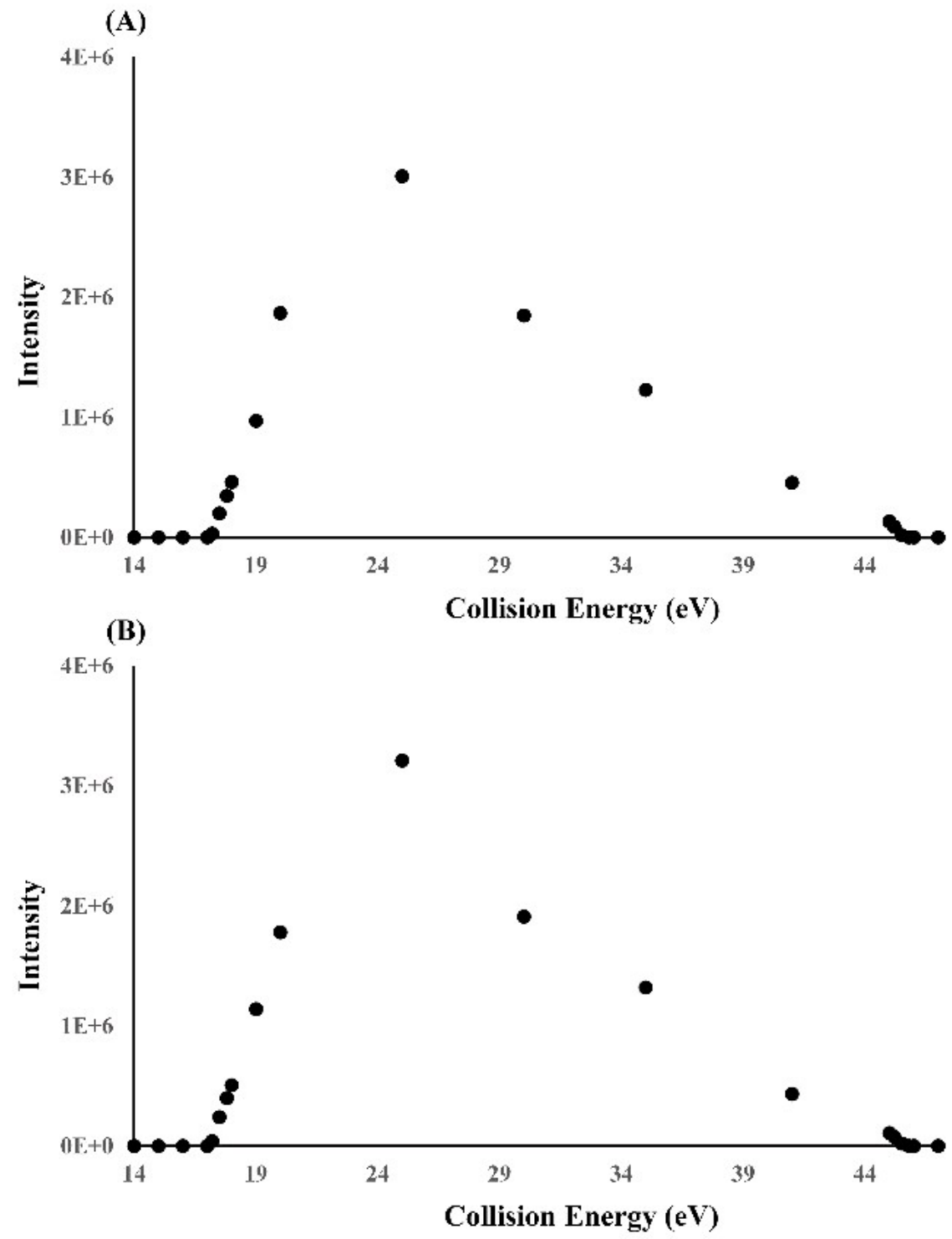

Fig. S4. Energy-resolved CID formation profile of the diagnostic B5 ion (m/z 666) for impurity 3 (A) and impurity 5 (B).

Table S1. Energy-resolved CID formation data of the diagnostic B5 ion (m/z 666)

Collision Energy(eV)	Impurity 3	Impurity 4	Impurity 5	Impurity 6
14	ND	ND	ND	ND
15	ND	ND	ND	ND
16	ND	ND	ND	ND
17	ND	ND	ND	ND
17.2	3.19E+04	ND	4.03E+04	ND
17.5	2.01E+05	ND	2.41E+05	ND
17.8	3.47E+05	ND	3.99E+05	ND
18	4.61E+05	ND	5.07E+05	ND
19	9.73E+05	ND	1.14E+06	ND
20	1.87E+06	ND	1.78E+06	ND
25	3.01E+06	ND	3.21E+06	ND
30	1.85E+06	ND	1.91E+06	ND
35	1.23E+06	ND	1.32E+06	ND
41	4.55E+05	ND	4.34E+05	ND
45	1.33E+05	ND	1.08E+05	ND
45.2	8.96E+04	ND	7.65E+04	ND
45.5	1.97E+04	ND	2.02E+04	ND
45.8	ND	ND	ND	ND
46	ND	ND	ND	ND

47	ND	ND	ND	ND

ND：Not Detected

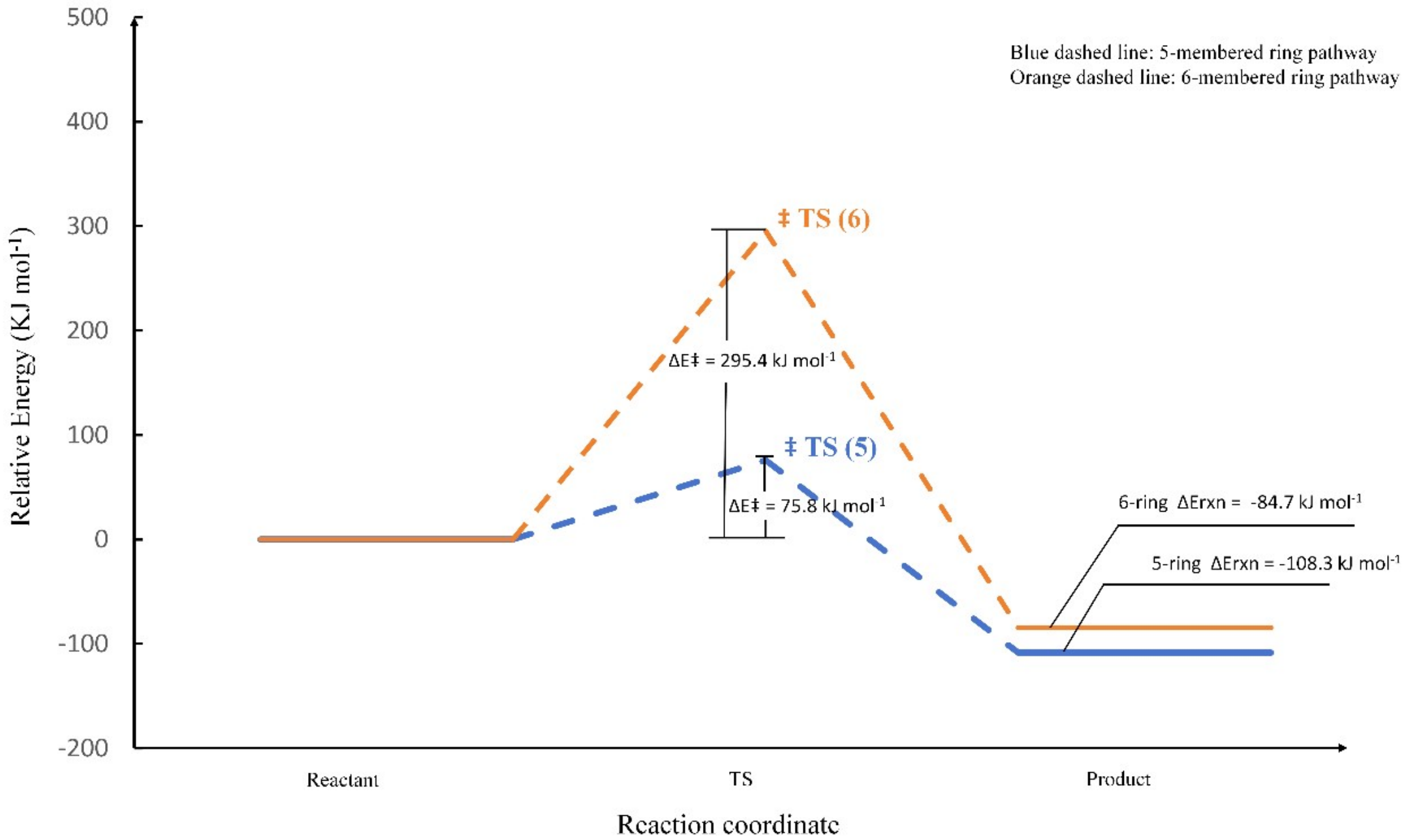

Fig. S5. Calculated energy profiles for the five-membered-ring (blue) and six-membered-ring (orange) cyclization pathways associated with the proposed gas-phase fragmentation mechanisms. Relative free energies are shown after zero-point energy (ZPE) correction.

Table S2. ZPE-corrected relative free energies of the optimized reactants, transition states, and products involved in the five-membered-ring and six-membered-ring cyclization pathways. Electronic energies including zero-point energy corrections (E + ZPE, Hartree) and the corresponding relative free energies are listed.

Structure	E + ZPE (Hartree)	Relative Energy ($kJ\ mol^{-1}$)
5-ring_R	-684.559981	0
5-ring_TS	-684.531102	75.82181
5-ring_P	-684.71727	-108.34651
6-ring_R	-684.517971	0.00000
6-ring_TS	-684.405477	295.35300
6-ring_P	-684.559238	-84.71701

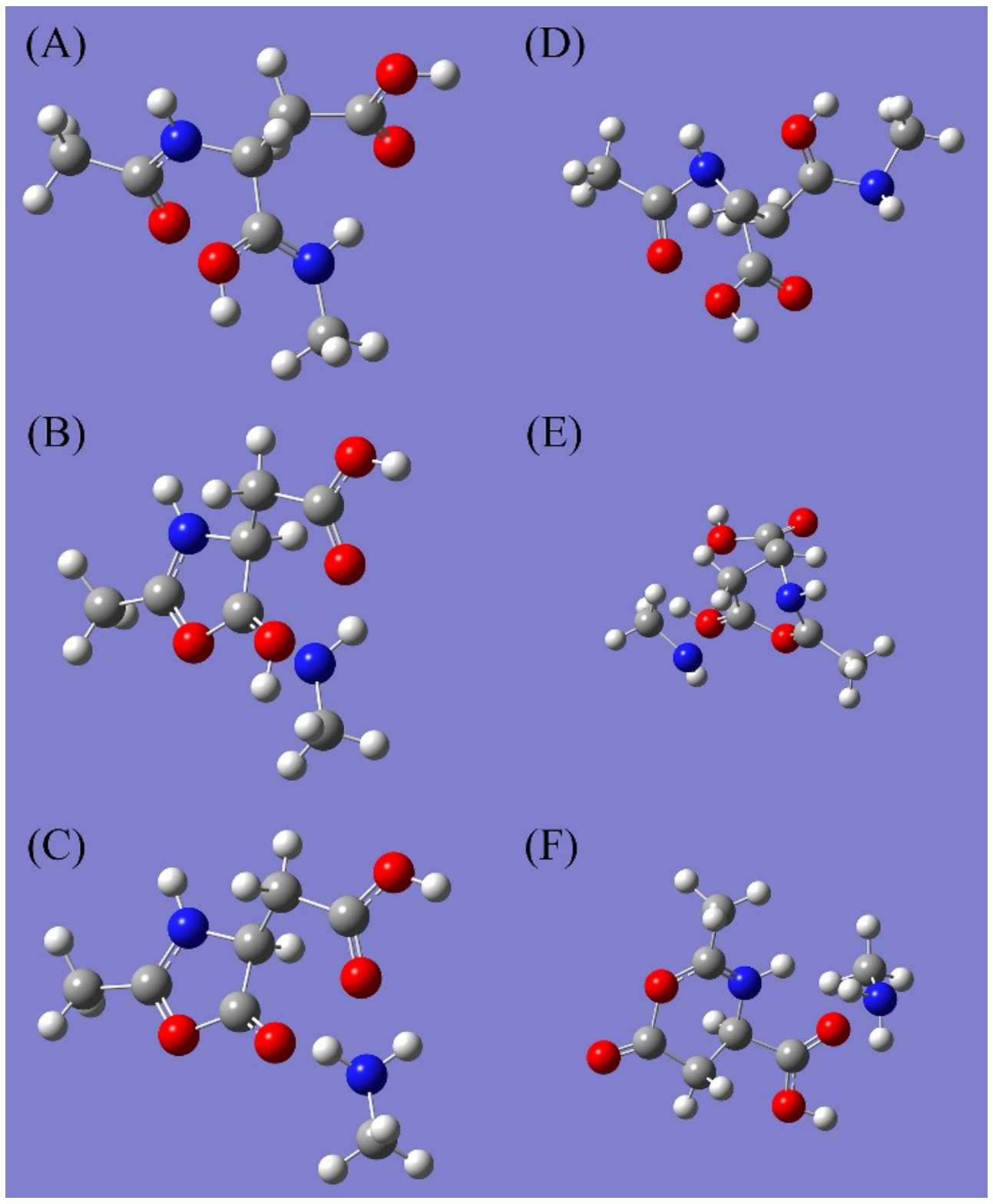

Fig. S6. Optimized structures obtained from DFT calculations for the proposed cyclization pathways. (A) Reactant, (B) transition state, and (C) product corresponding to the five-membered-ring cyclization pathway; (D) reactant, (E) transition state, and (F) product corresponding to the six-membered-ring cyclization pathway.

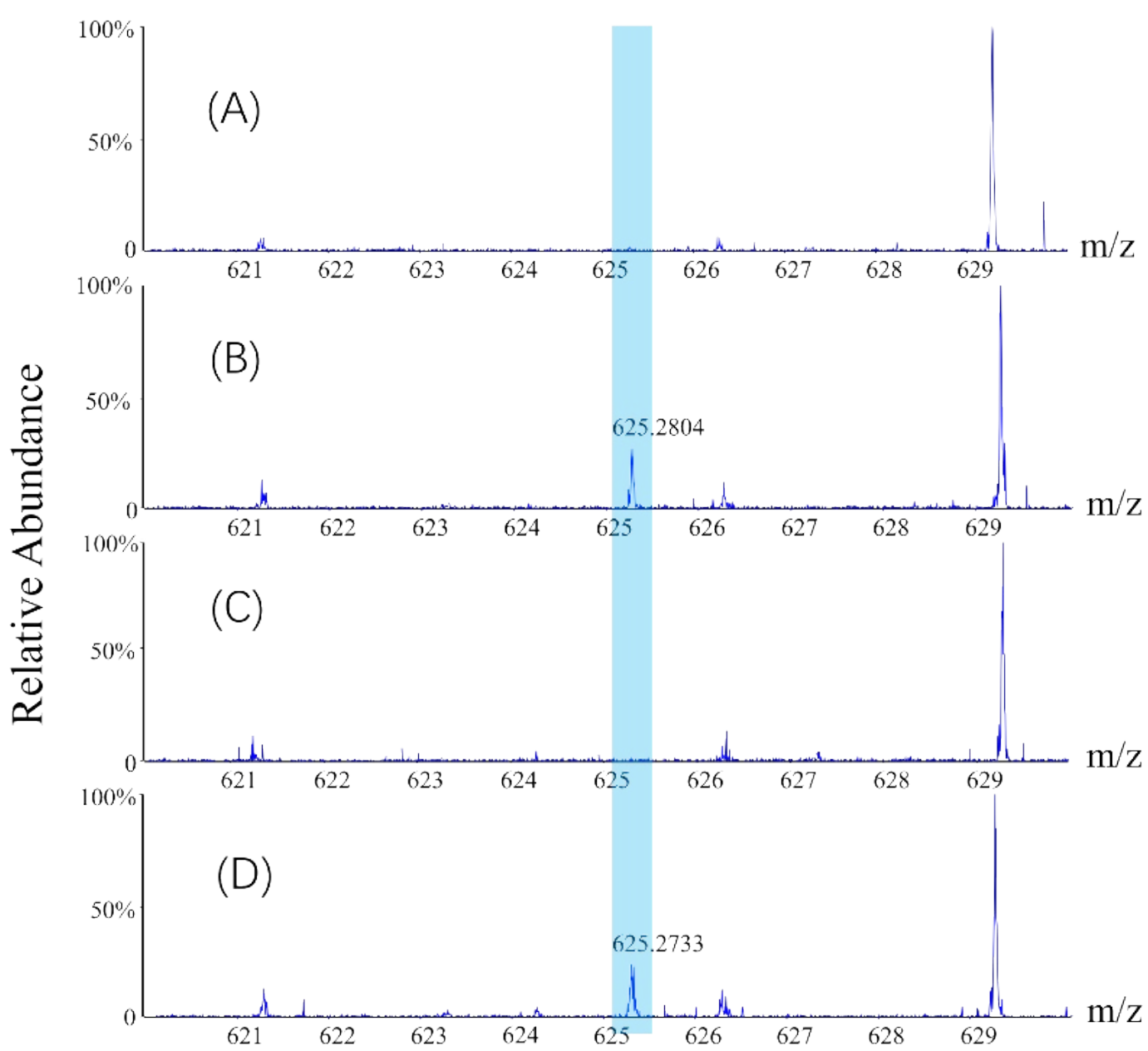

Fig. S7 EAD MS/MS spectra of the four hydrolytic impurities demonstrating the formation of the diagnostic c_5 +57.028 Da ion. This ion is clearly observed for Impurities 4 (B) and 6 (D), confirming the presence of isoAsp residues, whereas it is absent for Impurities 3 (A) and 5 (C) under identical experimental conditions.
